# Response of Tibetan Wild Barley Genotypes to Drought Stress and Identification of Quantitative Trait Loci by Genome-Wide Association Analysis

**DOI:** 10.3390/ijms20030791

**Published:** 2019-02-12

**Authors:** Mian Zhang, Man-Man Fu, Cheng-Wei Qiu, Fangbin Cao, Zhong-Hua Chen, Guoping Zhang, Feibo Wu

**Affiliations:** 1 Department of Agronomy, College of Agriculture and Biotechnology, Zijingang Campus, Zhejiang University, Hangzhou 310058, China; zhangmian7285@sxu.edu.cn (M.Z.); sunshineabigail@163.com (M.-M.F.); 3130100260@zju.edu.cn (C.-W.Q.); caofangbin@zju.edu.cn (F.C.); zhanggp@zju.edu.cn (G.Z.); 2 Institute of Applied Biology, Shanxi University, Taiyuan 030006, China; 3 Jiangsu Co-Innovation Center for Modern Production Technology of Grain Crops, Yangzhou University, Yangzhou 225009, China; 4 School of Science and Health, Hawkesbury Campus, University of Western Sydney, Penrith, NSW 2751, Australia; z.chen@uws.edu.au

**Keywords:** Diversity Array Technology (DArT) markers, *Hordeum vulgare* L. ssp. *vulgare*, drought stress, quantitative trait loci (QTL) mapping, genome-wide association (GWA)

## Abstract

Tibetan wild barley has been identified to show large genetic variation and stress tolerance. A genome-wide association (GWA) analysis was performed to detect quantitative trait loci (QTLs) for drought tolerance using 777 Diversity Array Technology (DArT) markers and morphological and physiological traits of 166 Tibetan wild barley accessions in both hydroponic and pot experiments. Large genotypic variation for these traits was found; and population structure and kinship analysis identified three subpopulations among these barley genotypes. The average LD (linkage disequilibrium) decay distance was 5.16 cM, with the minimum on 6H (0.03 cM) and the maximum on 4H (23.48 cM). A total of 91 DArT markers were identified to be associated with drought tolerance-related traits, with 33, 26, 16, 1, 3, and 12 associations for morphological traits, H^+^K^+^-ATPase activity, antioxidant enzyme activities, malondialdehyde (MDA) content, soluble protein content, and potassium concentration, respectively. Furthermore, 7 and 24 putative candidate genes were identified based on the reference Meta-QTL map and by searching the Barleymap. The present study implicated that Tibetan annual wild barley from Qinghai–Tibet Plateau is rich in genetic variation for drought stress. The QTLs detected by genome-wide association analysis could be used in marker-assisting breeding for drought-tolerant barley genotypes and provide useful information for discovery and functional analysis of key genes in the future.

## 1. Introduction

Drought is one of the major environmental stresses restricting crop productivity and occurs largely due to the effects of global climate change, the depletion of the underground water table, and erratic rainfall patterns [1,2]. The tolerance of crops to drought stress is a complex quantitative trait that involves a number of physiobiochemical processes at the cellular and organism levels during plant development [3]. Plants adapt to drought stress by developing a thicker cuticle and wax [4] and closing stomata to reduce water loss [5], promoting deeper root systems to increase water uptake [6], reducing high levels of reactive oxygen species (ROS) [7], and accumulating osmolytes to regulate cellular osmolality [8].

Barley (*Hordeum vulgare* L. ssp. *vulgare*) is one of the most widely cultivated cereal crops in the world and a suitable model crop for research into drought tolerance [9]. Tibetan annual wild barley (*Hordeum vulgare* L. ssp. *Spontaneum*) is considered a progenitor of cultivated barley, and its growth in the harsh environment of the Qinghai–Tibet Plateau has resulted in substantially greater genetic variation and stress tolerance than cultivated barley [10,11]. In a previous study, we evaluated the genetic diversity of cultivated and Tibetan wild barley using 20 genomic simple sequence repeat (SSR) and 49 expressed sequence tag SSR (EST-SSR) markers and identified 213 alleles [12]. The polymorphism information content (PIC), a value often used as an informative measure of a genetic marker in linkage studies, was 0.44 and 0.37 for the wild and cultivated barley, respectively. Thus, the Tibetan wild barley is likely to provide germplasm containing genes confer drought tolerance that can be introduced into cultivated barley to produce high-yielding cultivars with increasing yield stability under drought stress.

Genome-wide association (GWA) mapping, also known as linkage disequilibrium mapping, is an excellent molecular genetic analysis tool to detect marker-trait associations. Compared with the biparental mapping of quantitative trait loci (QTL), GWA has a higher mapping resolution and it is not limited to alleles segregating among the biparental populations [13]. GWA mapping has been conducted to successfully link molecular markers to important traits in rice [14], maize [15], wheat [16] and barley [17] and is being extended to other crops along with the development of high-throughput marker-genotyping and sequencing technologies [18]. Most of the reported QTLs for drought tolerance in barley are for yield and yield components under water-limited conditions [19,20]. Hu et al. [21] identified 913 quantitative trait nucleotides associated with 14 agronomic traits related to yield of barley using doubled haploid lines. Jabbari et al. [22] identified 167 marker trait associations for 11 yield components of modern European spring barley cultivars under drought stress. However, little information is available for QTLs associated with the morphological and physiological traits for Tibetan wild barley under drought stress.

A hydroponic experiment and a pot trial were undertaken to identify marker-trait associations for agronomic and physiological traits of Tibetan wild barley under drought stress. We evaluated the population structure, linkage disequilibrium (LD) decay, and phenotypic variation of Tibetan wild barley accessions to determine the associations between diversity array technology (DArT) markers and phenotypes relevant to drought tolerance. Moreover, we identified the major alleles and candidate genes for drought tolerance in Tibetan wild barley.

## 2. Results

### 2.1. Phenotypic Variation of Morphological Traits in Wild Barley

**Figure 1:**
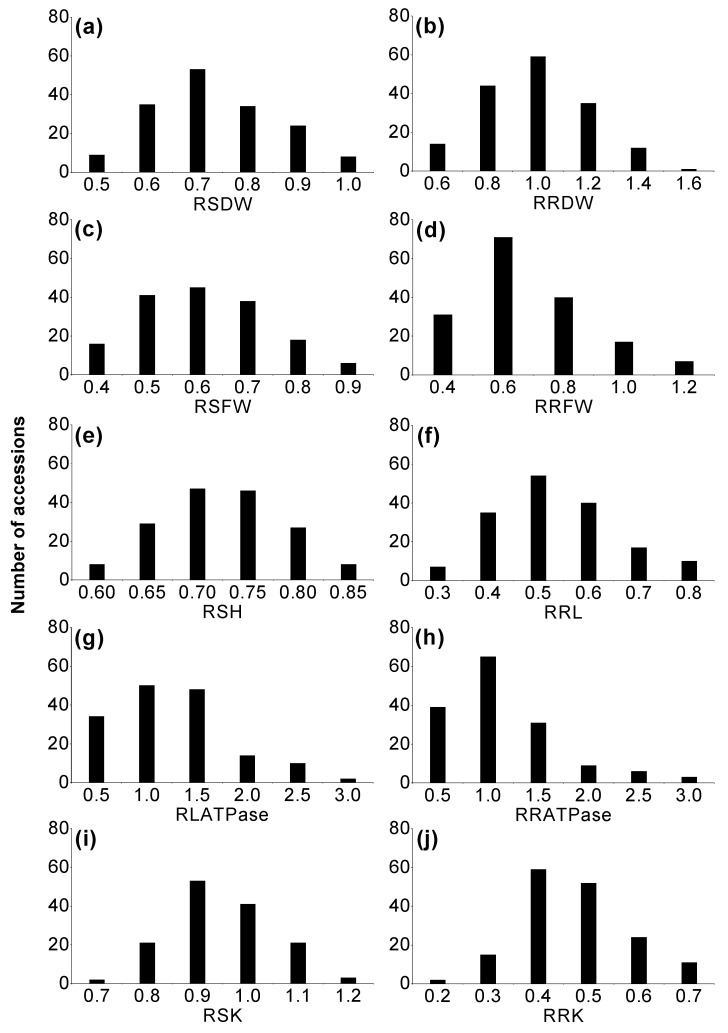
Frequency distribution of the relative value of different growth and physiological parameters of Tibetan wild barley plants under drought treatment in hydroponic experiment. Relative value of a given parameter was calculated as parameters under drought stress relative to control conditions (i.e., relative value = (parameter under drought stress)/(parameter under control)). (**a**) RSDW, relative shoot dry weight; (**b**) RRDW, relative root dry weight; (**c**) RSFW, relative shoot fresh weight; (**d**) RRFW, relative root fresh weight; (**e**) RSH, relative shoot height; (**f**) RRL, relative root length; (**g**) RLATPase, relative activity of leaf ATPase; (**h**) RRATPase, relative activity of root ATPase; (**i**) RSK, relative K concentration in shoots; (**j**) RRK, relative K concentration in roots.

**Figure 2:**
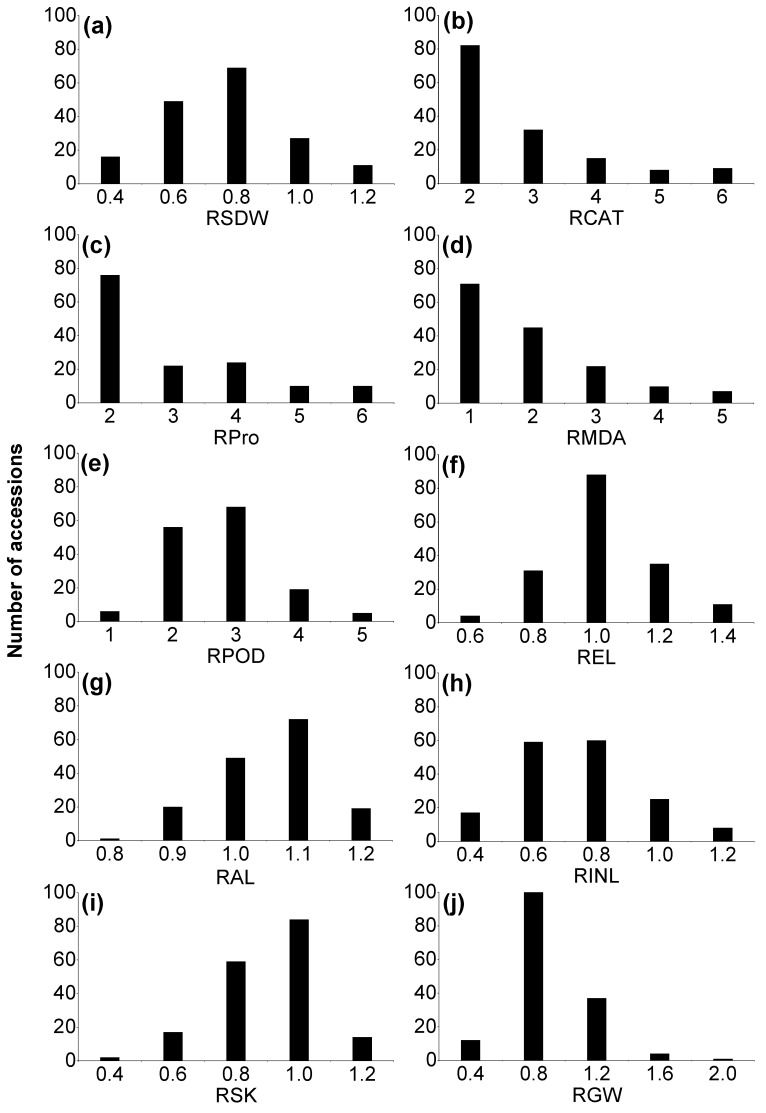
Frequency distribution of the relative value of different traits of Tibetan wild barley plants under drought treatment in pot experiment. Relative value of a given parameter was calculated as parameters under drought stress relative to control conditions (i.e., relative value = (parameter under drought stress)/(parameter under control)). (**a**) RSDW, relative shoot dry weight; (**b**) RCAT, relative catalase (CAT) activity in leaf; (**c**) RPro, relative soluble protein content in leaf; (**d**) RMDA: relative malondialdehyde (MDA) content in leaf; (**e**) RPOD, relative peroxidase (POD) activity in leaf; (**f**) REL: relative ear length; (**g**) RAL: relative awn length; (**h**) RINL: relative internode length below spike; (**i**) RSK, relative K concentration in shoots;(**j**) RGW: relative grain weight per spike.

The frequency distributions of the morphological parameters in the hydroponic experiment are shown in Figure 1. Large differences were found for relative shoot dry weight (RSDW) and relative root dry weight (RRDW) among the accessions, with values ranging from 0.42 to 0.98 and 0.42 to 1.48, respectively (Figure 1a,b). The root-to-shoot dry weight ratio (R: S) was also evaluated, with the relative values ranging from 0.63 to 2.12. Relative shoot height (RSH) and relative root length (RRL) ranged from 0.57 to 0.84 and 0.26 to 0.79, respectively (Figure 1c–f; Table 1). The results of correlation analysis showed that there were significant positive correlations between RSFW, RRFW, RSDW and RRDW (*p* < 0.01) (Table 2). RSH showed strong positive correlations with RSFW, RRFW, RSDW and RRDW, but RRL only significantly positively correlated with RSDW (*p* < 0.01). In the pot experiment, RSDW varied from 0.20 to 1.14 with an average of 0.66, which is consistent with that in the hydroponic experiment (Figure 2a). The relative values of awn length (RAL) and grain weight per spike (RGW) ranged from 0.78 to 1.20 and 0.10 to 1.77, respectively. There were significant positive correlations among RAL, RGW, relative ear length (REL), and relative internode length (RINL) (*p* < 0.01) (Figure 2f–h,j; Table 1 and Table 3).

### 2.2. Variation in H^+^K^+^-ATPase and Antioxidant Enzyme Activities, Malondialdehyde (MDA) and Soluble Protein Contents

In the hydroponic experiment, the relative values of H^+^K^+^-ATPase activity of leaves (RLATPase) and roots (RRATPase) ranged from 0.16 to 3.36 and 0.07 to 3.56, respectively (Figure 1g,h), which were significantly and positively correlated (*p* < 0.01). Furthermore, it is worth noting that RLATPase was significantly and positively correlated with RRDW (*p* < 0.05) (Table 2). 

In the pot experiment, the relative values of catalase (RCAT) and peroxidase (RPOD) activities, varied from 0.34 to 6.99 and 0.30 to 4.46, respectively (Figure 2b,e; Table 1). Large differences were also found in the relative values of soluble protein (RPro) and MDA contents (RMDA) among the accessions, ranging from 0.71 to 6.37 and 0.20 to 4.51, respectively (Figure 2c,d; Table 1). RPro also showed significant and positive causal links with RCAT, RMDA, and RSDW (Table 3). 

### 2.3. Phenotypic Variation in K^+^ Concentration in Wild Barley

In the hydroponic trial, 7 days of drought stress caused substantial variation in the relative values of the K^+^ concentration in shoots (RSK) and roots (RRK) among the accessions, ranging from 0.69 to 1.14 and from 0.21 to 0.68, averaged at 0.91 and 0.43, respectively (Figure 1i,j; Table 1). RRK correlated significantly and positively with RRFW and RRDW; in contrast, RSK had significantly negative correlation with RSDW (*p* < 0.01) (Table 2). 

In the pot experiment, there was also a large variation in RSK, ranging from 0.37 to 1.13 with a mean at 0.80 (Figure 2i) after 30 days of drought stress. RSK was also negatively correlated with RSDW (Table 3). RSK was also highly positively correlated with RPOD and RMDA (*p* < 0.01). 

### 2.4. Population Structure and Linkage Disequilibrium Decay

**Figure 3:**
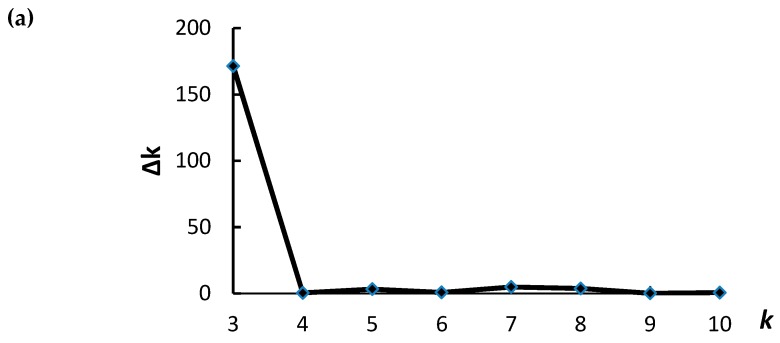

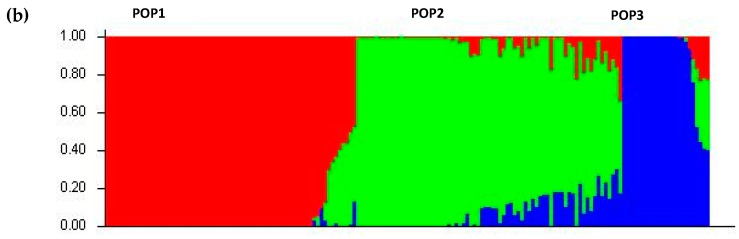
(**a**) The distribution of Δk, indicating the most appropriate cluster number (*k*) is three; (**b**) Population structure of 166 Tibetan wild barley genotypes at *k* = 3 based on genetic diversity detected by 777 DArT markers.

The population structure of the 166 barley accessions was analyzed using STRUCTURE and the 777 DArT markers. The most suitable number of subpopulations for association analysis was determined based on the highest Δk value = 3 (Figure 3a). The three subpopulations consisted of 69, 73 and 24 accessions (Figure 3b; Table S1). 

Linkage disequilibrium (LD) decay in the seven chromosomes of the wild barley accessions was deemed to have occurred when *r*^2^ ≤ 0.1. The results showed that the minimum LD decay distance is 0.03 cM on chromosome 6H and the maximum LD decay distance was 23.48 cM on chromosome 4H, with an average distance of 5.16 cM (Figure S1).

### 2.5. Identification of Loci Associated with Morphological Traits

In order to detect candidate loci for drought tolerance, a genome wide association analysis was performed using the 777 DArT markers and the traits determined under drought stress in both experiments. Based on a statistical significance level of *p* ≤ 0.001, a total of 91 marker-trait associations were detected in this study (Table 4 and Table 5), among which 33 markers were associated with morphological traits (Table 4). For RRDW, 11 associations were detected on chromosomes 1H, 2H, 3H, 4H and 7H and, these markers represented 9.8–15.6% of the phenotypic variation. The data from the pot experiment identified one marker (bpb-3653) on 2H associated with RSDW, explaining 9.4% of the phenotypic variation. In the hydroponic experiment, there were four and two associations with RSH and RRL, and these loci explained 9.1–10.5% and 8.9–12.3% of the phenotypic variation, respectively. Furthermore, 14 markers were associated with awn length (AL), and a major locus (bpb-0068) located at 66.50 cM on chromosome 3H contributed to 19.8% of the phenotypic variation. In addition, marker bpb-4184 on 2H was associated with both AL and internode length (INL) and explained 12.4% and 9.7% of the phenotypic variation, respectively.

### 2.6. Identification of Loci Associated with H^+^K^+^-ATPase and the Antioxidant Enzyme Activities, Malondialdehyde and Soluble Protein Contents

As shown in Table 5, there were 26 loci associated with leaf and root H^+^K^+^-ATPase activity under drought stress. Twenty-two associations with H^+^K^+^-ATPase were detected in root explaining between 11.2–23.2% of the phenotypic variation. Especially, markers bpb-1137 on 3H and bpb-3722 and bpb-9299 on 6H explained 23.2%, 22.5% and 20.8% of phenotypic variation for RRATPase, respectively (Table 5). It was noticeable that these three major loci and bpb-6884 on 3H were also associated with root H^+^K^+^-ATPase (RATPase) activity. Markers, bpb-3921 and bpb-8779, associated with RLATPase and leaf H^+^K^+^-ATPase (LATPase), respectively, were also linked to RRATPase (Table 5). 

With respect to the antioxidative system, 2 and 14 associations were detected for POD and CAT activity, respectively (Table 5). For CAT activity, the 14 loci explained from 9.3% to 26.4% of the phenotypic variation, among which three major loci, bpb-2304, bpb-7063 and bpb-7069 contributed to 26.4%, 25.7% and 25.7% of the phenotypic variation, respectively. 

Among the three markers associated with soluble protein content, a major locus on 1H (bpb-9957) explained 20.2% of the phenotypic variation. It is worth noting that three phenotypic parameters—RLATPase, RPro and RRK—were all associated with bpb-9957, suggesting that this marker may be significantly correlated with drought tolerance. There was only one marker, bpb-7723, associated with MDA content, explaining 12.8% of the phenotypic variation.

### 2.7. Identification of Loci Associated with K^+^ Concentration

For the shoot K^+^ concentration, four markers, bpb-3382, bpb-0689, bpb-3045 and bpb-9908, were associated with both RSK and shoot K^+^ concentration (SK), each contributing to 13.4–14.4% and 9.6–10.5% of phenotypic variation, respectively (Table 5). There were two closely linked associations (bpb-0910 and bpb-9957) located at 59.42 and 63.32 cM on 1H for root K^+^ concentration, explaining 9.1% and 7.6% of the phenotypic variation, respectively. In the pot experiment, two associations (bpb-8112 on 1H and bpb-1447 on 7H) were detected, explaining 9.2% and 9.4% of the phenotypic variation, respectively (Table 5).

## 3. Discussion

The present study reemphasizes the genetic diversity of Tibetan annual wild barley from the Qinghai–Tibet Plateau with potential genes and traits for drought stress tolerance. There were large differences in morphological traits, enzyme activities and K^+^ concentration, suggesting different mechanisms of drought tolerance among the accessions (Figure 1, Figure 2 and Figure 3). 

### 3.1. Quantitative Trait Loci (QTL) Associated with Morphological Traits under Drought Stress

Drought stress reduces shoot biomass of the barley accessions, but the mechanisms of drought response may be different in hydroponic and pot experiments. Similar reduction of RSDW in the hydroponic and pot experiments is in agreement with the findings of previous studies [3,23]. The results also showed that RRDW was higher than RSDW in the hydroponic experiment resulting in an increased root-to-shoot ratio under drought stress, indicating that a greater proportion of assimilated carbon was allocated to roots [24]. The significant positive correlation between RSDW and RRL (Table 2) suggests that deeper rooting mechanism may have resulted in greater shoot biomass and improved drought tolerance. A higher root-to-shoot ratio during drought was found to be combined with a relatively deeper distribution of roots [25]. A plant able to access water deep in soil at reduced metabolic cost will have superior productivity, because root construction and maintenance require metabolic investment [26]. 

The sequences of DArT markers associated with traits of barley under drought stress were aligned on Barleymap (http://floresta.eead.csic.es/barleymap/, accessed on 22th, April, 2017) to search the genes close to DArT markers, based on intervals of 5 cM (Table 6). The markers associated with root dry weight, bpb-9005 and bpb-3491, were close to two genes, *MLOC_59277* and *MLOC_74469*, both involved in lateral root primordium (LRP) protein. Sehgal et al. [27] indicated that the assimilate supply reduction under drought stress strongly influenced grain development, by decreasing assimilate production and mobilization to seeds in crops. In barley, 76% of the seed dry weight constitutes carbohydrates derived from the photosynthetic organs (lemma, palea and awn) of the spike [28] where awn is the major photosynthetic organ [29]. Previous studies revealed that the contribution of spike photosynthesis to grain-filling is greater during drought, as the flag leaves wilt under drought while the spike is more resilient and adapted to drought stress due to its better osmotic adjustment, high water use efficiency and delayed senescence [30,31,32]. The significant positive correlation (Table 3) between the awn length and grain weight per spike in this study further confirms that awn contributes to the photosynthesis and grain-filling of spikes during the reproductive stage of barley under drought stress [33].

### 3.2. QTL Associated with H^+^K^+^-ATPase under Drought Stress

The plasma membrane H^+^-ATPase plays important roles in plant development including powering the absorption and transport nutrients across membranes [34]. The function of H^+^-ATPase is to transport intracellular H^+^ and extracellular K^+^ across membranes by hydrolyzing ATP [35]. Under drought stress, increased H^+^-ATPase activity is an adaptive response of plants to prevent the direct or indirect dehydration of cell organelles [36]. The pH in the apoplast increases with the elevated H^+^-ATPase activity, and then ion channels and co-transporters are driven to transport the osmoregulation substances (e.g., K^+^, Cl^−^, organic acids) [37] into cells to decrease the cellular water potential. Gong et al. [38] demonstrated that drought stress can stimulate the activity of H^+^-ATPase by increasing the catalytic activity of its phosphatase domain. Our previous study [39] indicated that the drought tolerant Tibetan wild barley accessions XZ5 showed higher H^+^-ATPase activity under drought, low pH and Al combined stress. In this study, higher H^+^-ATPase and antioxidant enzymes activities was also observed in XZ5 with high tolerant to drought among the population. In addition, three markers bpb-4144, bpb-3722 and bpb-7399, associated with root H^+^K^+^-ATPase activity, were located close to 2, 7 and 2 genes on Barleymap (Table 6), among which there were 5 genes encoding ATP-binding cassette (ABC) transporters, 2 encoding ATPases, 2 potassium transporters, 1 potassium channel, and 1 ADP/ATP carrier protein. The marker bpb-9957 associated with root potassium content and leaf H^+^K^+^-ATPase activity, was close to two genes, *MLOC_37216* and *MLOC_18422*, involved in ABC transporter and phospholipid-transporting ATPase 3, respectively. However, further studies are required to analyze the function of these genes and their roles in drought tolerance of Tibetan wild barley.

### 3.3. QTL Associated with K^+^ Concentration under Drought Stress

There are many K^+^-dependent processes in plants, such as photosynthesis, osmoregulation and nutrition absorption [40,41,42]. Potassium ions can also improve plant tolerance to various abiotic stresses such as drought [41,43], NaCl [44,45] and waterlogging [46,47]. In the present study, the K^+^ concentration in most barley accessions decreased under drought stress, due to the impaired transpiration rate and the reduced membrane permeability [48]. Recent studies show that K^+^ efflux is important for sensing of ROS, induction of programmed cell death (PCD), and inhibition of energy-consuming biosynthesis [49,50]. Therefore, this may be another mechanism of potassium-regulated drought tolerance of Tibetan wild barley. Furthermore, this study identified two genes encoding K homology type 1, *AK366430* and *MLOC_5191.1*, which were located close to the marker associated with root potassium content, bpb-0910 (Table 6). Two potassium transporters and one potassium channel genes were identified to be associated with RRATPase.

### 3.4. QTL Associated with Peroxidase (POD) and Catalase (CAT) Activity under Drought Stress

There was considerable variation among barley accessions for activities of antioxidant enzymes (e.g., POD and CAT), the contents of MDA and soluble protein (Figure 2). The increased MDA content indicated serious membrane lipid peroxidation, which is consistent with the increased antioxidant enzyme activities needed to scavenge the high levels of ROS [7,41,51]. Under drought stress, the increase of soluble protein may result from the synthesis of highly hydrophilic protein for resistance of dehydration [2,52]. By searching the Barleymap using DArT marker sequence, six genes encoding peroxidase were identified close to the markers associated with POD and CAT activity. Besides, the gene *MLOC_78556* encoding high mobility group protein was close to the marker bpb-5403, associated soluble protein content (Table 6).

### 3.5. The Effect of Population Structure and Linkage Disequilibrium (LD) on the Association Analysis

Population structure analysis showed that three subpopulations are present in 166 Tibetan wild barley accessions. The grouping of the accessions into these subpopulations was in agreement with our previous study [12] using different markers, indicating the consistency in allocating the subpopulations. The presence of population stratification and an unequal distribution of alleles could result in non-functional and spurious associations [53]. Thus, the population structure was taken into account in this study. The average LD decay distance of 5.16 cM and the randomly distributed 777 DArT markers over the barley genome ensured that these markers cover the whole genome and are sufficient for genome wide association analysis. LD decayed more rapidly on chromosome 6H (0.03 cM) than 4H (23.48 cM), indicating that the results of GWA on 6H may provide higher resolution of fine mapping and gene discovery than those on 4H.

### 3.6. Comparison of Genome-Wide Association (GWA) Results with Reported Meta-QTL and Exploration of Candidate Genes

The previous meta-analysis of QTL associated with tolerance to abiotic stresses in barley identified 26 metaQTL (MQTL) and 29 candidate genes for traits associated with drought tolerance [19]. The consensus map based on the MQTL was selected as the reference map in this study. Twelve DArT markers identified in the present study located at similar positions to 10 MQTLs on the reference map (Table S2). Zhang et al. [54] also projected four MQTL for drought tolerance to the physical map of barley through meta-analysis.

The marker bpb-9957 localized at 63.32 cM on 1H was co-located to the MQTL D15 (61.60 cM, 1H). The NCBI blast results showed that the candidate gene for D15 was the homolog of the unigene *AtAGD8* in *Arabidopsis thaliana*. *AtAGD8* (ADP-ribosylation factor GTPase-activating protein domain), and its homologous gene in rice, *OsAGAP*, is essential for plant development and growth [55,56]. Similarly, the three markers at 94.9 cM on 1H, bpb-6911, bpb-9121 and bpb-4144, associated with relative root H^+^K^+^-ATPase, was co-located to the MQTL1H.4 (102.2 cM, 1H) for drought tolerance identified by Zhang et al. [54]. MQTL1H.4 included the candidate gene ATP-dependent Clp protease ATP-binding subunit ClpX1 (*CLPX*). The marker bpb-3382 (66.24 cM, 1H), bpb-9005 (67.88cM, 1H) and bpb-5334 (67.88cM, 1H) were located at less than 3 cM away from the MQTL D16 (69.4cM, 1H) on 1H. The candidate gene *ELIP2* (Early Light-Inducible protein2) in barley is related to the regulation of chlorophyll concentration in thylakoids and the redox homeostasis under photooxidative stress and water deficit conditions [57,58]. The marker bpb-8779 (77.41 cM, 2H) was close to the MQTL D4, which was localized at 76.77 cM on 2H. There was one homolog of the candidate gene for D4 in *Arabidopsis*, *AtPQL1* (PsbQ-like protein1). Previous studies suggested that PQL1 and PQL2 might function in the chloroplast NAD(P)H dehydrogenase (NDH) complex, which functioned in PSI cyclic electron flow [59]. Consequently, the barley ortholog of *AtPQL1* may be involved in the response of plants to drought stress by influencing the photosynthetic system.

There was another homolog of the candidate gene for D4 in *Arabidopsis*, *AtZEP* (zeaxanthin epoxidase). The enzyme zeaxanthin epoxidase (ZEP) catalyzes the conversion of zeaxanthin to violaxanthin, a key reaction for ABA (abscisic acid) biosynthesis, which is important for acclimation to environmental stress, in particular drought. Drought stress can lead to an increase of ZEP in roots and to a degradation of ZEP in leaves, with similar variations in ABA [60]. Teng et al. [61] revealed that the ABA biosynthesis-related gene *OsZEP* in rice was up-regulated under drought stress. The marker bpb-2548 at 48.32 cM on 3H was co-located to MQTL D10 (45.77 cM, 3H). The candidate gene for D10 was the homolog of *AtPLDα1* (Phospholipase Dα1), which played roles in cellular regulation and signal transduction [62]. As to drought tolerance, *AtPLDα1* was implicated in mediating the ABA-regulated stomatal movement and ABA-induced gene expression, as the inhibition of *AtPLDα1* diminished stomatal closure induced by ABA or drought and increased water loss in *Arabidopsis* [63]. The marker bpb-6611 (60.55 cM, 4H) was located similarly to MQTL D14 at 57.34 cM on 4H. The candidate homolog gene, *AtPCK1* (*phosphoenolpyruvate carboxykinase*), is specifically expressed in guard cells and trichomes of the leaf in *Arabidopsis*. Previous studies showed that *pck1* mutant plants have increased stomatal conductance and reduced drought tolerance, indicating that *AtPCK1* was also involved in stomatal movement under drought stress [64]. 

## 4. Materials and Methods 

### 4.1. Plant Materials

A total of 166 Tibetan wild barley accessions, sourced from the barley germplasm collection at Huazhong Agricultural University (Wuhan, China) were used. The hydroponic and pot experiments were conducted on Zijingang Campus, Zhejiang University, Hangzhou, China, in 2015 and both had complete randomized block designs with three replicates.

### 4.2. Hydroponic Experiment

In order to determine the traits related with roots, without damaging the root system, the hydroponic experiment was conducted. The traits of leaves from the hydroponic system can also be the validation of the following pot experiment. Seeds were surface sterilized in 2% H_2_O_2_ for 30 min, rinsed in distilled water and soaked for 3 h at 20 °C in the dark, and then germinated on moist filter paper in an incubator with a 20 °C/14 °C day / night temperature regime. Ten-day-old uniform seedlings were transplanted into 45 L containers covered with a polystyrol plate with 48 evenly spaced holes (two plants per hole). The containers were placed in a greenhouse which had the same conditions with the climate outside, except for a canopy to keep the rain off, and were filled with 40 L of basal nutrient solution (BNS) as described in Wu et al. [51]. The nutrient solution was continuously aerated with pumps and renewed once a week. The pH of the BNS was regulated to 5.8–6.0 with NaOH or HCl. On the 7th day after transplanting, two treatments were imposed on the plants: (1) control—the roots of plants were kept in the nutrient solution throughout the experiment; (2) drought stress—the plants were raised so that their roots were exposed to air for 6 h (9 am–3 pm) daily for 7 days [65]. Compared with the polyethylene-glycol (PEG) or other osmotics simulated drought stress, the method in this study can avoid the influence of osmotics on the root traits. After 7 days of treatment, the shoots and roots were assessed and sampled separately. After measuring shoot height, root length and fresh weight, the topmost fully expanded leaves were sampled and stored at −80 °C. Then, the shoots and washed roots were oven-dried for 72 h at 70 °C to determine their dry weights.

### 4.3. Pot Experiment

On the 7th day after watering the air-dried soil in 45 L containers with BNS, seeds were sown in soil from the field experimental station of Zijingang campus. One week after emergence, six uniform seedlings were retained in each pot. At the two-leaf stage, two treatments were applied: (1) control—the soil in the pots was kept moist [soil moisture content (SMC) of 35 ± 5%] throughout the experiment; (2) drought stress—stop watering plants for 30 days until the SMC reduced to 4%. The soil moisture content was measured using an HH2 Moisture Meter (Delta-T Devices, Cambridge, UK) every second day. The topmost fully expanded leaves of two seedlings and the shoots of another two seedlings were sampled under both control and drought stress of 4% SMC. For drought stress, the two remaining seedlings were treated repeatly with the cycle of drought (4% SMC) and recovery (35 ± 5% SMC) until the maturation stage, while under control condition, plants were well-watered until harvest. The ears from the last developing node (top node) were harvested and the awn, internode, ear lengths, and grain weight were measured.

### 4.4. Determination of H^+^ K^+^-ATPase Activity

The 0.3 g topmost fully-expanded leaves sampled from the hydroponic experiment were ground with 50 mM Tris-HCl (pH = 7.4). The activity of H^+^ K^+^-ATPase was determined using an activity assay kit (Jiancheng Bio Co., Nanjing, China) according to the manufacturer’s instructions.

### 4.5. Determination of Antioxidant Enzyme Activity, and Malondialdehyde (MDA) and Soluble Protein Contents

The 0.3 g samples of topmost fully-expanded leaves were collected from control and treatment plants from the pot experiment 30 days after the commencement of the drought treatment. The tissue samples were homogenized in 8 mL of 50 mM phosphate buffer (PBS, pH 7.8), then centrifuged at 10,000 g and 4 °C for 15 min, then the supernatants were used for measurement. MDA content, and catalase (CAT, EC 1.11.1.6) and peroxidase (POD, EC 1.11.1.7) activities were then determined according to Zhang [66]. The soluble protein content was estimated by the Coomassie brilliant blue staining method [67].

### 4.6. Determination of K^+^ Concentration

Dried shoots and roots from the two experiments were weighed and ashed at 500 °C for 12 h. The ash was digested with 5 mL 30% HNO_3_, and then diluted with 5 mL deionized water. The K^+^ concentrations in the digests were then determined using flame atomic absorption spectrometry (Model AA-6300, Shimadzu, KyotoJapan).

### 4.7. Population Structure and Linkage Disequilibrium (LD)

Genetic diversity was analyzed in the 166 wild barley accessions using 777 DArT markers [68] with only a minor allele frequency >0.03. The genetic polymorphism, which was determined using the 777 DArT markers, was used to assess any population structure using the STRUCTURE program (v. 2.3.3, http://pritch.bsd.uchicago.edu/structure.html, accessed on 14th October 2016) [69] in an admixture model, setting clusters (*k*) from 1 to 15 and performing ten independent runs of 100,000 Markov Chain Monte Carlo iterations. The largest value of statistic Δk indicated the number of clusters (k) [70].

The linkage disequilibrium (LD) of the seven chromosomes was estimated using the 777 DArT markers by TASSEL software (v. 3.0, Buckler lab, New York, NY, USA, http://www.maizegenetics.net) [71]. For each chromosome, the genetic distances between pairs of alleles were regressed against the squared values allele frequency correlations and LD decay curves were fitted and plotted using Origin Pro (v. 8.0, Origin Lab Corporation, Wellesley Hills, Wellesley, MA, USA).

### 4.8. Genome-Wide (GWA) Association Analysis

Association analysis between the 777 DArT markers and phenotypes of the wild barley accessions under drought treatment was performed using TASSEL v. 3.0. The Q matrix, estimating group membership coefficients for each accession, was extracted from STRUCTURE (*k* = 3) to correct for population structure in the association mapping. The kinship, pair-wise relationship matrix, was estimated using SPAGeDi [72] and was used as a cofactor in the regression model. Therefore, the GWA analysis was performed using a mixed linear model, considering both the Q matrix and kinship as cofactors. The Benjamini–Hochberg false discovery method was used to determine if a locus was associated with a trait. Locus with *p* ≤ 0.001 was considered to be associated with a particular trait.

### 4.9. Statistical Analysis

Data presented are the average of three independent replicates. Relative values were calculated as the ratio of values under drought stress to those in the control conditions. Correlation analyses were performed to find association among phenotypic traits. Analysis of variance (ANOVA) was performed to determine differences in morphological and physiological traits using Duncan’s Multiple Range tests (DMRT) at *p* < 0.05 and *p* < 0.01. These analyses were performed using SPSS (v.16.0, Hearne Software, IBM, Stanford, CA, USA).

## 5. Conclusions

In conclusion, there were large genotypic variations among Tibetan wild barley accessions for drought tolerance-related traits. Genome-wide association analysis detected 91 QTL and 31 putative candidate genes for further studies on gene discovery and functional analysis and marker-assistant breeding. However, confirmation studies are required to assess the allele effects more precisely and develop stable drought-tolerant barley varieties through genetic transformation and association studies.

## Figures and Tables

**Table 1:** The relative values of growth and physiological parameters of 166 Tibetan wild barley genotypes in hydroponic and pot experiments, expressed as drought stress relative to control.

Experiments	Traits	Minimum	Maximum	Average	CV (%)	Between Genotypes
Hydroponic	RSDW	0.42	0.98	0.69	18.0	**
	RRDW	0.42	1.48	0.90	23.6	**
	RSFW	0.26	0.96	0.57	23.3	**
	RRFW	0.27	1.16	0.57	34.2	**
	RSH	0.57	0.84	0.71	8.4	**
	RRL	0.26	0.79	0.49	24.7	**
	RLATPase	0.16	3.36	1.06	61.8	**
	RRATPase	0.07	3.56	0.86	75.9	**
	RSK	0.69	1.14	0.91	10.7	**
	RRK	0.21	0.68	0.43	23.8	**
Pot	RSDW	0.20	1.14	0.66	29.9	**
	RPOD	0.30	4.46	2.18	36.6	**
	RCAT	0.34	6.99	2.02	74.7	**
	RMDA	0.20	4.51	1.48	71.6	**
	RPro	0.71	6.37	2.28	60.7	**
	REL	0.52	1.38	0.93	17.1	**
	RAL	0.78	1.20	1.01	8.4	**
	RINL	0.29	1.33	0.65	30.8	**
	RSK	0.37	1.13	0.80	18.6	**
	RGW	0.10	1.77	0.71	37.4	**

Note: CV: Coefficient of variation. **, Significant at the 0.01 probability levels between genotypes. Traits of Hydroponic Experiment: RSDW, relative shoot dry weight; RRDW, relative root dry weight; RSFW, relative shoot fresh weight; RRFW, relative root fresh weight; RSH, relative shoot height; RRL, relative root length; RLATPase, relative activity of leaf ATPase; RRATPase, relative activity of root ATPase; RSK, relative K concentration in shoots; RRK, relative K concentration in roots. Traits of Pot Experiment: RSDW, relative shoot dry weight; RPOD, relative peroxidase (POD) activity in leaf; RCAT: relative catalase (CAT) activity in leaf; RMDA: relative malondialdehyde (MDA) content in leaf; RPro, relative soluble protein content in leaf; REL: relative ear length; RAL: relative awn length; RINL: relative internode length below spike; RGW: relative grain weight per spike.

**Table 2:** The correlation coefficient among the parameters of growth and physiological traits of 166 Tibetan wild barley genotypes in the hydroponic experiment.

	RSDW	RRDW	RSFW	RRFW	RLATPase	RRATPase	RSH	RRL	RSK	RRK
RSDW	1.000									
RRDW	0.479 **	1.000								
RSFW	0.896 **	0.375 **	1.000							
RRFW	0.400 **	0.730 **	0.362 **	1.000						
RLATPase	0.110	0.168 *	−0.033	0.142	1.000					
RRATPase	0.067	0.090	0.031	−0.023	0.554 **	1.000				
RSH	0.440 **	0.189 *	0.548 **	0.303 **	−0.034	0.058	1.000			
RRL	0.155 *	−0.059	−0.095	−0.200	−0.280 **	0.019	0.006	1.000		
RSK	−0.409 **	0.029	−0.078	0.072	−0.117	−0.077	−0.093	0.085	1.000	
RRK	0.067	0.171 *	0.185 *	0.303 **	0.066	0.024	0.170 *	-0.007	−0.010	1.000

Note: * and **, Significant at the 0.05 and 0.01 probability levels between genotypes, respectively.

**Table 3:** The correlation coefficient among the parameters of growth and physiological traits of 166 Tibetan wild barley genotypes in the pot experiment.

	RSDW	RSK	RPOD	RCAT	RMDA	RPro	RAL	RINL	REL	RGW
RSDW	1.000									
RSK	−0.112	1.000								
RPOD	−0.012	0.210 **	1.000							
RCAT	0.067	0.051	−0.066	1.000						
RMDA	−0.073	0.259 **	−0.075	−0.032	1.000					
RPro	0.163 *	0.126	−0.133	0.410 **	0.384 **	1.000				
RAL	0.122	−0.050	−0.135	−0.036	0.039	0.012	1.000			
RINL	0.081	−0.056	−0.187 *	0.005	0.157 *	0.223 **	0.329 **	1.000		
REL	0.118	−0.058	−0.255 **	0.027	0.129	0.153	0.515 **	0.551 **	1.000	
RGW	0.202 **	−0.138	−0.224 **	−0.059	0.126	0.172 *	0.396 **	0.289 **	0.554 **	1.000

Note: * and **, Significant at the 0.05 and 0.01 probability levels between genotypes, respectively.

**Table 4:** List of DArT markers with significant marker-trait association of morphological traits.

Traits	Marker	Chromosome	Distance (cM)	−log_10_ (p)	r^2^ (%)
RRDW	bPb-9005	1H	67.88	4.72	13.0
	bPb-4481	2H	18.81	4.37	13.1
	bPb-0827	2H	86.41	4.34	14.2
	bPb-0775	2H	140.87	4.34	13.0
	bPb-2203	3H	35.93	5.16	15.6
	bPb-8013	4H	86.69	3.37	13.3
	bPb-6096	4H	96.78	3.33	9.8
	bPb-3491	7H	100.50	4.44	9.9
	bPb-6384	7H	100.50	4.53	13.6
	bPb-3506	7H	101.20	4.53	13.6
	bPb-8037	7H	160.68	4.40	13.2
RSH	bPb-1127	1H	57.23	3.35	9.9
	bPb-9336	3H	100.76	3.48	10.3
	bPb-8419	3H	153.55	3.11	9.1
	bPb-3375	6H	122.08	3.57	10.5
RL	bPb-8935	1H	118.95	4.14	12.3
RRL	bPb-9908	7H	111.69	3.77	8.9
RSDW	bPb-3653	2H	108.05	3.05	9.4
AL	bPb-2976	1H	54.01	4.11	12.9
	bPb-5334	1H	67.88	4.25	13.4
	bPb-5444	2H	26.24	3.38	10.5
	bPb-4184	2H	119.90	3.95	12.4
	bPb-1264	3H	5.97	3.80	9.6
	bPb-0068	3H	66.50	6.09	19.8
	bPb-6611	4H	60.55	4.27	13.5
	bPb-9820	4H	142.09	4.16	13.1
	bPb-1485	5H	81.35	4.07	12.8
	bPb-2835	5H	81.35	3.56	11.1
	bPb-8022	5H	101.34	3.33	10.4
	bPb-8553	5H	120.44	4.09	12.9
	bPb-4457	7H	3.02	4.41	13.9
	bPb-8051	7H	78.22	3.25	10.1
INL	bPb-4184	2H	119.90	3.15	9.7

**Table 5:** List of DArT markers with significant marker-trait association of physiological traits.

Traits	Marker	Chromosome	Distance (cM)	−log_10_ (p)	*r*^2^ (%)
RRATPase	bPb-3217	1H	40.53	3.34	12.4
	bPb-6911	1H	94.90	4.07	15.3
	bPb-9121	1H	94.90	4.08	15.3
	bPb-4144	1H	94.90	3.06	11.3
	bPb-4877	2H	47.38	3.03	11.2
	bPb-8779	2H	77.41	3.03	11.2
	bPb-1593	2H	149.44	3.11	11.5
	bPb-8255	2H	149.44	3.85	14.4
	bPb-6884	3H	0.98	4.09	15.3
	bPb-3025	3H	9.88	3.05	11.3
	bPb-1137	3H	10.20	6.00	23.2
	bPb-2548	3H	48.32	4.68	17.7
	bPb-9299	6H	14.35	5.44	20.8
	bPb-3921	6H	38.16	4.35	16.4
	bPb-3722	6H	68.53	5.83	22.5
	bPb-7399	7H	94.41	3.13	11.6
RLATPase	bPb-9957	1H	63.32	3.37	8.1
	bPb-3921	6H	38.16	3.37	10.1
	bPb-4369	6H	74.34	3.11	9.4
RATPase	bPb-8081	1H	116.46	3.01	12.7
	bPb-6884	3H	0.98	4.09	17.5
	bPb-1137	3H	10.20	4.46	19.2
	bPb-9299	6H	14.35	3.17	13.4
	bPb-3722	6H	68.53	4.35	18.7
	bPb-6607	6H	84.64	3.48	14.8
LATPase	bPb-8779	2H	77.41	3.06	9.2
RSK	bPb-3382	1H	66.24	4.73	14.4
	bPb-0689	2H	157.09	4.72	14.4
	bPb-3045	4H	65.34	4.42	13.4
	bPb-9908	7H	111.69	4.72	14.4
RRK	bPb-0910	1H	59.42	3.10	9.1
	bPb-9957	1H	63.32	3.25	7.6
SK	bPb-3382	1H	66.24	3.22	9.6
	bPb-0689	2H	157.09	3.25	9.7
	bPb-3045	4H	65.34	3.49	10.5
	bPb-9908	7H	111.69	3.29	9.8
MDA	bPb-7723	2H	163.34	4.00	12.8
POD	bPb-8737	2H	108.72	3.07	9.5
RPOD	bPb-5755	2H	133.29	3.35	10.6
RPro	bPb-9957	1H	63.32	6.72	20.2
	bPb-5403	7H	159.05	3.06	7.8
	bPb-6701	7H	159.19	3.06	7.8
SK (soil)	bPb-1447	7H	78.22	3.21	9.4
	bPb-8112	1H	140.85	3.14	9.2
RCAT	bPb-0994	2H	113.25	3.43	9.3
	bPb-4601	2H	157.00	5.69	16.1
	bPb-1857	3H	0.98	3.43	9.4
	bPb-9599	3H	149.85	3.40	9.3
	bPb-0347	3H	175.25	4.89	13.6
	bPb-7069	3H	178.60	8.66	25.7
	bPb-7063	3H	178.60	8.66	25.7
	bPb-8580	5H	8.49	4.98	13.9
	bPb-3792	5H	45.58	5.07	14.2
	bPb-5584	5H	54.10	5.28	14.8
	bPb-2304	6H	136.06	8.89	26.4
	bPb-7146	6H	137.76	4.40	12.2
	bPb-1621	6H	137.76	5.17	14.5
	bPb-0783	7H	160.68	4.71	13.1

**Table 6:** The function description of genes close to DArT markers by searching Barleymap.

Trait	Marker	Chromosome	cM	Gene	cM	Description
RRDW	bPb-9005	1H	54.39	*MLOC_59277*	52.51	Lateral root primordium (LRP) protein-related
	bPb-3491	7H	89.14	*MLOC_74469*	89.14	Lateral root primordium (LRP) protein-related
RRATPase	bpb-4144	1H	87.87	*MLOC_13794*	90.3	ATP-binding cassette transporter
				*MLOC_58493*	90.3	ABC (ATP-binding) family transporter
	bpb-3722	6H	54.89	*MLOC_68594*	52.83	Potassium transporter
				*MLOC_46910*	53.6	AAA-type ATPase family protein
				*MLOC_57716*	53.61	ATPase 7B; Copper-transporting ATPase 2
				*MLOC_10111*	53.9	Potassium channel
				*MLOC_72084*	54.82	ADP, ATP carrier protein 1
				*MLOC_34488*	54.89	ABC transporter ATP-binding protein
				*MLOC_5994*	55.03	ATP-binding cassette transporter
	bpb-7399	7H	84.56	*MLOC_52035*	84.57	ATP-binding cassette transporter
				*MLOC_13204*	89.14	Potassium transporter
RRK RLATPase	bpb-9957	1H	47.83	*MLOC_37216*	46.6	ABC transporter ATP-binding protein
				*MLOC_18422*	47.83	Phospholipid-transporting ATPase 3
RRK	bPb-0910	1H	46.81	*AK366430*	48.09	K Homology type 1
				*MLOC_5191.1*	52.48	K Homology type 1 subgroup
POD	bPb-8737	2H	98.52	*MLOC_25978*	98.52	Peroxidase 60, putative
RPOD	bPb-5755	2H	119.76	*MLOC_66485*	119.33	Peroxidase 16
				*MLOC_15491*	124.77	Peroxidase
RCAT	bPb-4601	2H	144.12	*MLOC_65477*	146.53	Peroxidase 10
				*MLOC_54893*	146.92	Peroxidase 12
	bPb-2304	6H	117.55	*MLOC_55199*	117.55	Peroxidase superfamily protein
RPro	bPb-5403	7H	138.22	*MLOC_78556*	140.86	High mobility group protein

## Supplementary Materials

Supplementary materials can be found at http://www.mdpi.com/1422-0067/20/3/791/s1.
