# Supplementary material for: Response of Tibetan Wild Barley Genotypes to Drought Stress and Identification of Quantitative Trait Loci by Genome-Wide Association Analysis
Source: Int J Mol Sci. 2019 Feb 12;20(3):791. doi: 10.3390/ijms20030791 (PMC6387302; doi:10.3390/ijms20030791)
Supplement: Supplementary file 1 [file ijms-20-00791-s001.pdf]

International Journal of
Molecular Sciences

MDPI

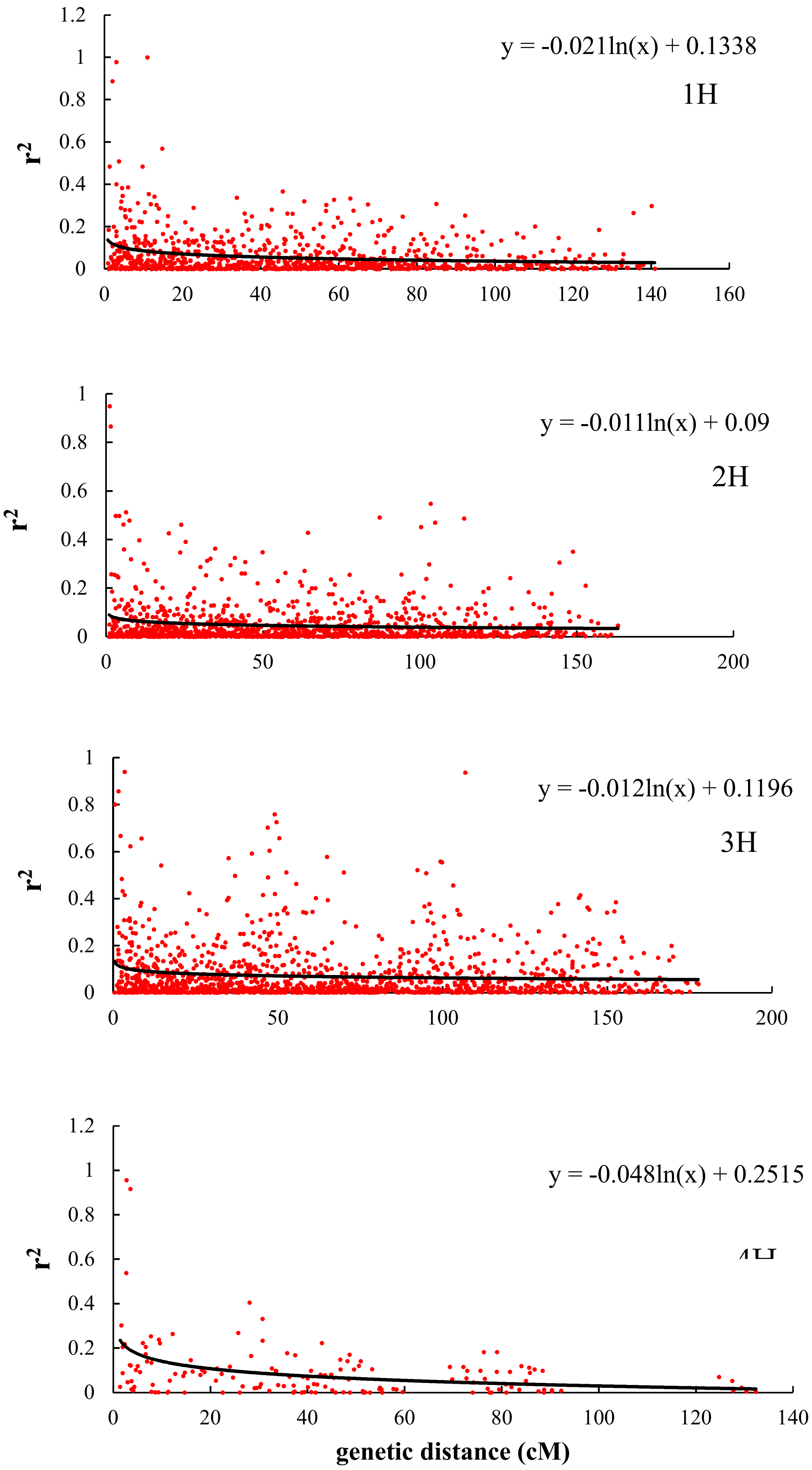

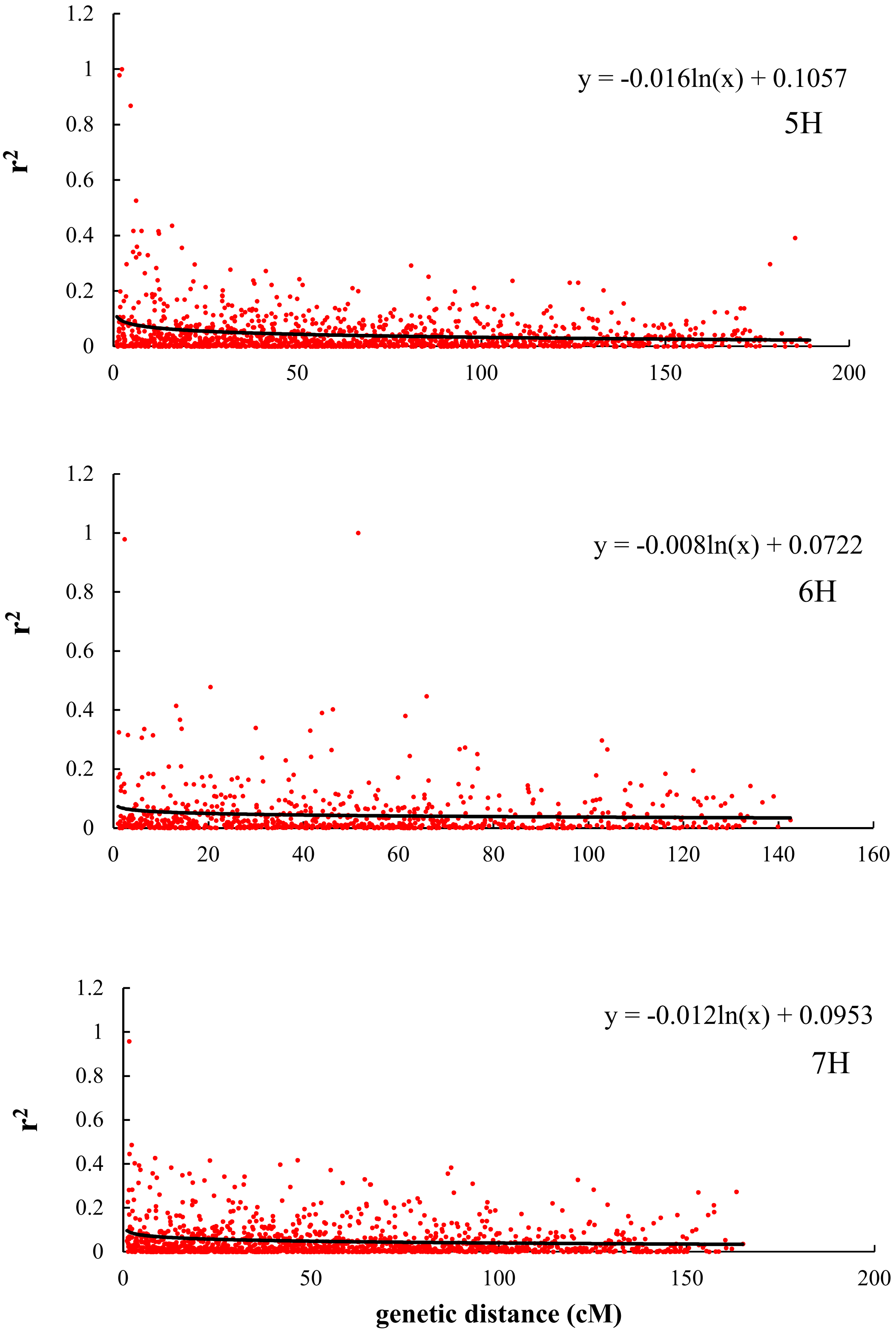

Fig. S1 Decay of linkage disequilibrium (LD) of the seven chromosomes of Tibetan wild barley genotypes.

Table S1. Distribution of 166 Tibetan wild barley genotypes in the three subpopulations.

Subpopulation	Accession number								
POP1	XZ101	XZ104	XZ106	XZ108	XZ109	XZ111	XZ112	XZ115	XZ116
	XZ117	XZ118	XZ121	XZ123	XZ124	XZ125	XZ127	XZ130	XZ131
	XZ139	XZ142	XZ143	XZ145	XZ150	XZ154	XZ156	XZ158	XZ160
	XZ161	XZ162	XZ163	XZ164	XZ165	XZ166	XZ167	XZ169	XZ186
	XZ26	XZ35	XZ37	XZ39	XZ41	XZ42	XZ51	XZ53	XZ54
	XZ57	XZ58	XZ65	XZ67	XZ68	XZ69	XZ71	XZ72	XZ75
	XZ77	XZ78	XZ79	XZ81	XZ82	XZ87	XZ88	XZ89	XZ90
	XZ91	XZ94	XZ96	XZ97	XZ98	XZ99			
POP2	XZ10	XZ100	XZ103	XZ105	XZ107	XZ11	XZ110	XZ12	XZ120
	XZ126	XZ128	XZ129	XZ132	XZ135	XZ14	XZ144	XZ146	XZ149
	XZ15	XZ151	XZ152	XZ153	XZ155	XZ159	XZ16	XZ17	XZ171
	XZ172	XZ173	XZ175	XZ176	XZ177	XZ178	XZ18	XZ182	XZ183
	XZ184	XZ185	XZ187	XZ188	XZ19	XZ2	XZ20	XZ22	XZ24
	XZ25	XZ27	XZ28	XZ29	XZ3	XZ30	XZ31	XZ32	XZ33
	XZ34	XZ38	XZ4	XZ43	XZ49	XZ50	XZ52	XZ55	XZ6
	XZ62	XZ63	XZ7	XZ70	XZ8	XZ83	XZ85	XZ9	XZ92
	XZ93								
POP3	XZ114	XZ119	XZ122	XZ134	XZ136	XZ137	XZ138	XZ140	XZ141
	XZ170	XZ179	XZ180	XZ36	XZ45	XZ5	XZ59	XZ61	XZ64
	XZ66	XZ73	XZ74	XZ80	XZ84	XZ86			

Table S2. MQTL associated with drought tolerance on consensus map (Li et al,2013)

MQTL	Chr.	Position on Chr. (cM)	Flanking markers of the position	Traits under abiotic stress
D1	7H	79.46	E40M48-205-E41M32-698	WSC100, RWC, PH
D4	2H	76.77	E38M55-223-E36M48-400	L2L, SL, FD, SID, RER, GY, RLE
D5	2H	83.44	E36M62-495-MWG865	WSC100, WSC, L2L, RER
D7	2H	124.35	MSU21-GBM1200	Chl, Fv/Fm, Fv, Fm, Fo
D8	2H	140.18	E42M40-644-ABG316D	Chl, Fv/Fm, Fv, Fm
D10	3H	45.77	E37M33-239-E37M33-238	REG, TGW, PH, DMA
D14	4H	57.34	E33M61-440-E42M55-506	WSC100, TGW
D15	1H	61.60	E40M32-198-E35M55-88	RWC
D16	1H	69.40	E36M49-58-GBM1153	SeP, RWC
D22	6H	68.65	E40M32-409-E40M47-187	REG, PH, RLE

Chl, ChlorophyII content; DMA, days to maturity; FD, date of flowering; F_m, maximal fluorescence; F_o, initial fluorescence; F_v, variable fluorescence; F_v/F_m, maximum poteitial quantum; GY, grain yield; L2L, second leaf length; PH, plant height; REG, regrowth rate; RER, leaf relative elongation rate; RLE, root length; RWC, relative water content; SeP, seed per plant; SID, date of spike initiation;

SL, spikes per line; TGW, thousand grain weight; WSC, water soluble carbohydrate concentration; WSC100, WSC at 100 % RWC.
